# Immune Cells in Subretinal Wound Healing and Fibrosis

**DOI:** 10.3389/fncel.2022.916719

**Published:** 2022-06-10

**Authors:** Manon Szczepan, María Llorián-Salvador, Mei Chen, Heping Xu

**Affiliations:** ^1^The Wellcome-Wolfson Institute for Experimental Medicine, School of Medicine, Dentistry & Biomedical Sciences, Queen’s University Belfast, Belfast, United Kingdom; ^2^Aier Institute of Optometry and Vision Science, Changsha, China

**Keywords:** retina, inflammation, innate immunity, adaptive immunity, age-related macular degeneration, macular fibrosis, proliferative vitroretinopathy

## Abstract

The subretinal space is devoid of any immune cells under normal conditions and is an immune privileged site. When photoreceptors and/or retinal pigment epithelial cells suffer from an injury, a wound healing process will be initiated. Retinal microglia and the complement system, as the first line of retinal defense, are activated to participate in the wound healing process. If the injury is severe or persists for a prolonged period, they may fail to heal the damage and circulating immune cells will be summoned leading to chronic inflammation and abnormal wound healing, i.e., subretinal or intraretinal fibrosis, a sight-threatening condition frequently observed in rhematogenous retinal detachment, age-related macular degeneration and recurrent uveoretinitis. Here, we discussed the principles of subretinal wound healing with a strong focus on the conditions whereby the damage is beyond the healing capacity of the retinal defense system and highlighted the roles of circulating immune cells in subretinal wound healing and fibrosis.

## Introduction

Fibrosis is the formation of an abnormal amount of fibrous tissue in an organ as the result of dysregulated inflammation or wound healing. A variety of stimuli such as tissue injury, infection, autoimmune and allergic responses or radiation can trigger fibrosis (Wynn, 2008). Despite the large diversity of noxious signals, studies have reported that ischemia, abnormal angiogenesis, and chronic inflammation play an important role in the development and progression of fibrosis. When tissue suffers from injuries, the damaged cells release alarmins which recruit innate immune cells to remove the dead cells and repair the damage. However, if the injury is severe or persists, the wound healing process fails, and the tissue-educated innate immune cells summon adaptive immune cells for assistance (Matzinger and Kamala, 2011). The innate and adaptive immune systems may remove threats and tissue heals without fibrosis or scarring; they may also fail to solve the problem and the tissue is filled with, or even replaced by inflammatory fibrovascular membrane characterized by accumulation of various immune cells, new blood vessels, myofibroblasts and extensive deposition of Extracellular Matrix (ECM) proteins. During this process, the type of immune cells that infiltrate the tissue and their functions will determine the fate of the affected tissue. However, tissues are not simply passive recipients of immune protection but are active participants in their own defense (Matzinger and Kamala, 2011). Therefore, when tissues suffer from an insult, first, they must decide whether or not to call for assistance from the circulating immune system. They will then decide which immune cells should be summoned and what functions the immune cells will do. The type of immune cells and their released mediators vary in different tissues and under different conditions. The retina, particularly the subretinal space, is an immune privileged (IP) site. When damage occurs, such as in age-related macular degeneration (AMD), rhegmatogenous retinal detachment (RRD), or retinal penetrating injury, healing and repair can be very different from other tissues.

Here, we discuss the principles of the immune response to severe and/or persistent damages in the subretinal space, with a particular focus on the innate and adaptive immune cells and the mediators that may lead to the development of subretinal fibrosis. The majority of subretinal fibrosis develops secondary to neovascularization in AMD (nAMD) and RRD. In nAMD, the fibrotic lesion is located in the macula, therefore, is often called macular fibrosis. However, clinically, macular fibrosis includes pre-retinal macular fibrosis (also known as epiretinal membrane, retinal pucker) and subretinal macular fibrosis. The aetiologies of pre-retinal and subretinal macular fibrosis are different. To avoid any confusion, this article only discusses subretinal/intraretinal wound healing and fibrosis.

## Wound Healing and Fibrosis in the Subretinal Space

### Subretinal Space – An Immune Privileged Site

The subretinal space refers to the interface between the neuroretina and RPE/choroid where the adherence between neuroretina and RPE cells is relatively weak. It is considered an IP site and is devoid of any immune cells under normal physiological conditions. The IP is achieved by the physical barrier (i.e., the blood-retina-barrier, BRB), the lack of lymphatic system, and the immunological barrier i.e., the immune suppressive properties of retinal neurons and RPE cells (Forrester et al., 2008). The physical barrier includes the inner BRB (iBRB) formed by tight junctions between retinal endothelial cells and the outer BRB (oBRB) formed by tight junctions between RPE cells. The oBRB regulates the passage of solutes and nutrients from the choroid to the retina and prevents the leakage of macromolecules and harmful agents into the retina (Cunha-Vaz et al., 2011). Subretinal injury often leads to oBRB damage and the development of inflammatory or degenerative conditions, such as nAMD, RRD, or diabetic retinopathy (Smith et al., 1992; Cunha-Vaz et al., 2011). The iBRB may also be affected during subretinal injury likely due to injury-mediated oxidative stress and inflammation (Toris and Pederson, 1985).

The immunological barrier in the subretinal space is achieved by RPE and photoreceptor cells. RPE cells contribute to the establishment of IP state in the subretinal space due to their constitutive expression of CD95 ligand, known to be expressed in immune privilege tissues (Wenkel and Streilein, 2000). In addition, RPE cells produce various immune regulators that can induce effector T cells apoptosis (Lau and Taylor, 2009) or convert them into regulatory T cells (Kawazoe et al., 2012). RPE cells can also modulate macrophage complement expression at the retina-choroidal interface. For example, they can upregulate the expression of C1 inhibitor (C1INH) in infiltrating macrophages (Luo et al., 2018). Under disease conditions, activated RPE cells can release a range of pro- and anti-inflammatory factors (Zamiri et al., 2006). Photoreceptors also express various immune regulators such as CD47 and CD59 (Liu et al., 2020). Thus, RPE cells together with photoreceptors tightly regulate the microenvironment of subretinal space and maintain its IP state. When damage occurs, they will decide which immune cells to recruit and guide them to do what they are supposed to do in the subretinal space. However, if the damage is severe, photoreceptors may die and RPE cells may undergo epithelial-to-mesenchymal transition (EMT) leading to the loss of IP.

### Wound Healing in the Subretinal Space

**FIGURE 1:**
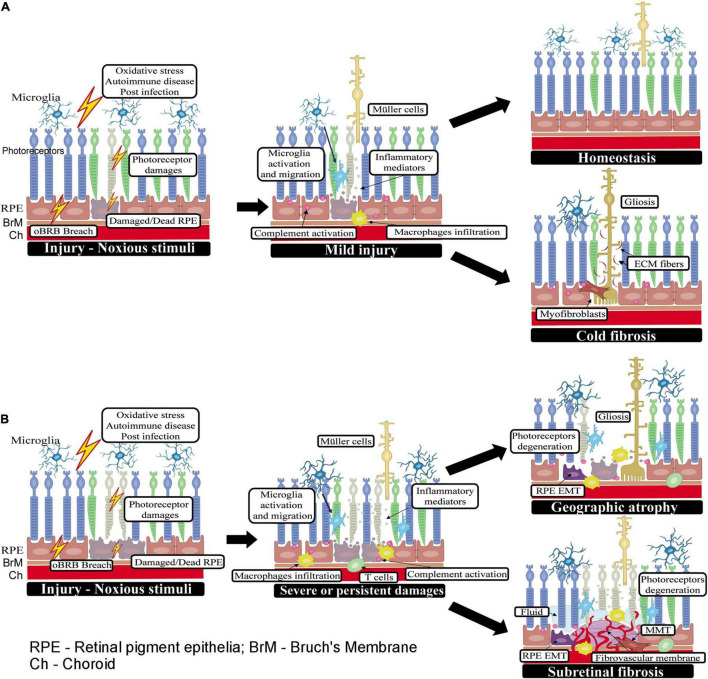
Wound healing in the subretinal space. **(A)** When the initial insult is mild or one-off, the injury can be promptly rectified by retinal innate immune system (may also be assisted by infiltrating innate immune cells) and the subretinal space returns to homeostasis. If the injury causes a significant number of photoreceptor loss, Müller cells will be activated (may also be transdifferentiated into myofibroblast). Muller cells, myofibroblasts along with ECM proteins produced by them will fill the space left by dead cells forming gliosis without ongoing inflammation (or cold fibrosis). **(B)** When the insult to photoreceptors or RPE cells persists or is severe, retinal immune system, circulating innate and adaptive immune cells may all participate in the healing process. If the subretinal damage does not involve the ingrowth of new blood vessels, such as in GA and RRD, the damaged photoreceptor/RPE cells may be replaced by active Müller glia (i.e., gliosis) or myofibroblasts transdifferentiated from other cells such as RPE cells or macrophages. In RRD-induced PVR, the wound healing response leads to excessive ECM deposition and the development of subretinal fibrotic membrane; whereas, in GA, the wound is filled with active Muller glia and infiltrating immune cells without excessive ECM deposition and damage area becomes atrophic. In nAMD, new blood vessels are a part of the pathology, and the healing process is accompanied by continued immune cell infiltration and excessive deposition of ECM around the diseased blood vessels and eventually, the development of fibro-vascular membrane.

A wound healing response in the subretinal space can be triggered by photoreceptor or RPE damage caused by multiple factors such as a breach in the oBRB, oxidative stress, post-infection and autoimmune response (e.g., autoimmune chorioretinitis), retinal detachment of rhegmatogenous origin (Idrees et al., 2019) or following administration of gene or cell therapy reagents into the subretinal space, etc. (Planul and Dalkara, 2017; Jin et al., 2019). The stressed RPE cells and/or photoreceptors will summon innate immune cells (i.e., microglia and macrophages) to clear the damage through phagocytosis as well as by releasing various inflammatory mediators (e.g., chemokines and cytokines) (Tonade et al., 2017; Detrick and Hooks, 2019). The complement system may be activated to promote the clearance of apoptotic cells through C3b-mediated opsonization. Retinal cells, including photoreceptors and RPE, are known to express complement components (Anderson et al., 2010; Xu and Chen, 2016; Liu et al., 2020). Subretinal microglia and macrophage accumulation and complement activation have been observed in normal aging (Xu et al., 2008; Ma et al., 2013), light-induced retinal degeneration (Rutar et al., 2011; Sennlaub et al., 2013) and various models of AMD (Combadière et al., 2007; Little et al., 2020b). In addition, RPE cells can act as a scavenger alongside the macrophages/microglia by phagocytosing debris (Friedlander, 2007). Müller cells may also be activated to participate in retinal repair. If the initial insult is cleared and the dead cells are removed, the innate immune response may heal the injury and the subretinal space returns to homeostasis (Figure 1A). However, if the injury causes a significant number of photoreceptor loss, active Müller cells along with ECM proteins produced by them will fill the space left by dead cells forming gliosis without ongoing inflammation (or cold fibrosis, see definition below) (Figure 1A).

If the insult to photoreceptors or RPE cells is severe or persists, adaptive immune cells may be recruited to the subretinal space. For example, aged Nrf2 knockout mice fed with high-fat diet developed RPE degeneration akin to dry AMD, which was accompanied by subretinal accumulation of MHC-II^+^ microglia, γδT cells and FoxP3^+^ regulatory T cells (Zhao et al., 2014b). In the meantime, Müller cells, particularly, those right on top of or near the damaged photoreceptor/RPE area may also be activated. In addition to their roles in maintaining retinal structure, neuroprotection and detoxification (Bringmann et al., 2006), Müller cells are critically involved in retinal wound healing and repair (Bringmann et al., 2009; Bringmann and Wiedemann, 2012). They can participate in retinal wound healing by secreting pro-/anti-angiogenic cytokines such as VEGF, Fibroblast Growth Factor-2 (FGF-2), Tumor Necrosis Factor-α (TNF-α) (Bringmann et al., 2006), Pigment Epithelium Derived Factor (PEDF), Transforming Growth Factor-ß (TGF-ß), and thrombospondin-1 (Eichler et al., 2004) and be an important source of collagen I-VII, IX, and XI (Ponsioen et al., 2008). Müller cells are also believed to be dormant resident progenitor cells that can repair retinal neurons (Karl and Reh, 2010; Liu et al., 2013a).

During subretinal would healing, RPE cells can undergo EMT and acquire macrophage- or fibroblast-like phenotype depending on the microenvironment that they are exposed to. Specifically, RPE cells tend to adopt fibroblast properties when the environment is rich in collagen and fibronectin but can switch to a macrophage-like phenotype in the presence of vitreous or photoreceptor debris (Grierson et al., 1994). Multiple pathways (e.g., TGF-β, Wnt, miRNA, oxidative stress/Nrf2, etc.) are known to play a role in EMT by RPE and this topic has been reviewed extensively recently by others (Yang et al., 2015; Shu et al., 2020; Zhou et al., 2020; Blasiak et al., 2021). However, the molecular cues that guide RPE cells transdifferentiating into macrophage- or fibroblast-like phenotypes remain to be fully elucidated. A classical wound healing has three phases: inflammation, proliferation, and remodeling, with the aim to clear dead cells/debris, heal and stabilize the wound. But if the damage is severe or persists for a prolonged period, wound healing and cell death may co-exist in the subretinal space leading to significant overlaps in the three phases. Infiltrating innate and adaptive immune cells, the complement system and retinal resident cells (e.g., Müller cells, RPE cells etc.) all participate in the healing process. When there is an increasing demand for the removal of dead cells and debris in the subretinal space, RPE cells may adapt to a macrophage-like phenotype through EMT. During the proliferation and healing stages, RPE cells may transdifferentiate into fibroblast-like cells to fill the space left by dead cells and participate in retinal remodeling.

The subretinal space is devoid of blood vessels. If the subretinal damage does not involve the ingrowth of new blood vessels, such as in RRD, the damaged photoreceptor/RPE cells may be replaced by active Müller cells (i.e., gliosis) or myofibroblasts transdifferentiated from Müller glia, RPE cells or macrophages. The subretinal membranes in PVR are reported to constitute multiple types of cells, including fibroblasts, RPE cells, Müller cells, infiltrating macrophages, CD4^+^ and CD8^+^ T cells (Charteris et al., 1993). In nAMD, new blood vessels are a part of the pathology, and the healing process is accompanied by continued immune cell infiltration and excessive deposition of ECM around the diseased blood vessels and eventually, the development of fibro-vascular membrane (Figure 1B). Immunohistochemistry studies revealed complement deposition and immune cell infiltrations in subretinal fibro-vascular membrane from nAMD patients (Grossniklaus et al., 2005; Little et al., 2020a). To differentiate the scars that are well-settled and contain only fibroblasts from the ones with active inflammation and contain both myofibroblasts and immune cells, Adler et al. (2020) defined the former as “cold fibrosis” and the later as “hot fibrosis”.

### Subretinal Fibrosis

Subretinal fibrosis is the end stage of various eye diseases including RRD (Pastor et al., 2016) and nAMD (Daniel et al., 2014), recurrent uveoretinitis (Kim et al., 1987), proliferative diabetic retinopathy (Roy et al., 2016), or subretinal neovascularization secondary to high myopia (Montero and Ruiz-Moreno, 2010). When the lesion is located in the macula, it is often called “macular fibrosis.” PVR is a major cause of retinal detachment surgery failure in RRD patients. Vitreous hemorrhage is known to be a risk factor for PVR (Duquesne et al., 1996). In nAMD, macular fibrosis stabilizes the neovascular membrane leading to non-responsiveness to the anti-VEGF treatment (Daniel et al., 2014; Ishikawa et al., 2016). Risk factors of nAMD-related macular fibrosis include initial worse visual acuity, persistent damage to RPE and the outer layers of the neuronal retina, a longer duration between disease onset and treatment and hemorrhage (Little et al., 2018; Teo et al., 2020). In addition to nAMD, choroidal neovascularization (CNV) also occurs in pathologic myopia (Ohno-Matsui et al., 2021) and recurrent uveoretinitis (Kim et al., 1987) and these patients are normally younger than nAMD patients. Interestingly, the CNVs in child and adolescent myopic patients are less likely to progress into macular fibrosis compared to those in nAMD. In fact, their CNV can regress spontaneously, and the regression is often accompanied by macular and choroidal atrophy but not fibrosis (Hayashi et al., 2010; Rishi et al., 2013). This suggests that old age increases the risk of subretinal fibrosis. RPE cells in the aging eye undergo significant cytoskeleton reorganization (Tarau et al., 2019) and are multinucleated and have impaired wound healing capacity (Chen et al., 2016b). The increased risk of subretinal fibrosis in the elderly may be related to RPE senescence and impaired wound healing.

The underlying mechanism of subretinal fibrosis is poorly defined although inflammation is believed to play an important role (Chen and Xu, 2015). A low-grade inflammation (para-inflammation) exists in the aging retina and RPE/choroid (Xu et al., 2009), which may favor a profibrotic response during subretinal wound healing. The risk factors of PVR and macular fibrosis in nAMD are indicatives of either severe insults to the macula or prolonged/sustained tissue damage, which will likely induce an inflammatory response that constitutes a variety of innate and adaptive immune cells. Activation of these immune cells creates a microenvironment that recruits and activates fibroblasts in the subretinal space, particularly when the oBRB is damaged such as in nAMD.

## Immune Cells in Subretinal Fibrosis

**FIGURE 2:**
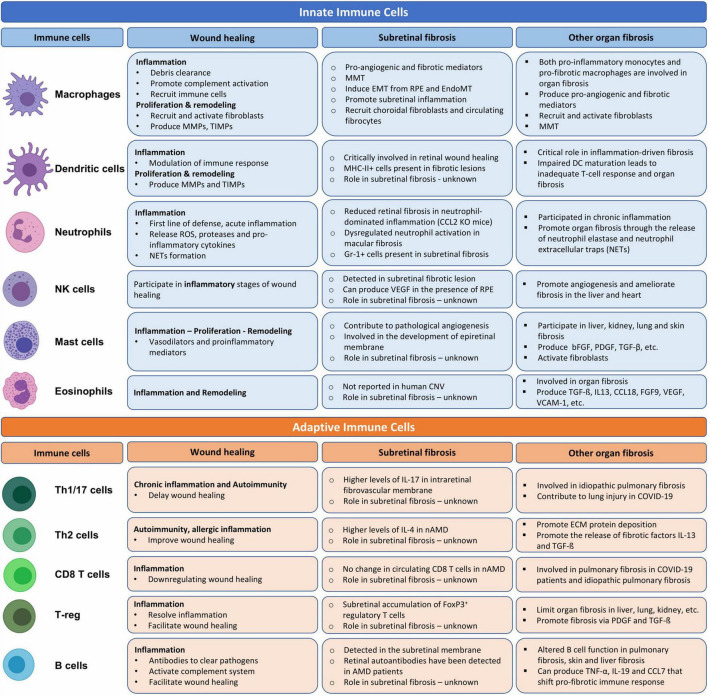
The role of innate and adaptive immune cells in wound healing, organ and subretinal fibrosis. Cells were adopted from BioRender.com.

After injury, a timely inflammation is essential to eliminate harmful stimuli and initiate wound healing. The initial inflammatory response is dominated by innate immune cells such as neutrophil, monocytes, and macrophages (Oberyszyn, 2007). Prompt resolution of the inflammation will facilitate tissue repair and the wound heals with “cold fibrosis.” However, if the inflammation fails to resolve, chronic inflammation will follow leading to further tissue damage and progressive fibrosis. Both the innate and adaptive immune cells participate in chronic inflammation. In this section, we discuss the role of innate cells and adaptive immune cells in subretinal wound healing and fibrosis (Figure 2).

### Innate Immune Cells

#### Macrophages

Subretinal phagocyte accumulation has been observed in the normal aging retina (Xu et al., 2008; Lad et al., 2015), after light damage (Sennlaub et al., 2013; Karlen et al., 2018) and is related to defective RPE immunomodulation (Xu et al., 2009). Although the source of subretinal phagocytes in various models of retinal degeneration (i.e., macrophages vs. microglia) differs (Karlen et al., 2018; Yu et al., 2020), infiltrating macrophages are believed to play a critical role in the laser-induced CNV and its related subretinal fibrosis (Sakurai et al., 2003; Tsutsumi et al., 2003; Little et al., 2020a). Both pro-inflammatory monocytes and pro-fibrotic and alternatively activated macrophages are reported to be involved in organ fibrosis including in severe COVID-19 patients (Page et al., 2012; Wendisch et al., 2021).

**FIGURE 3:**
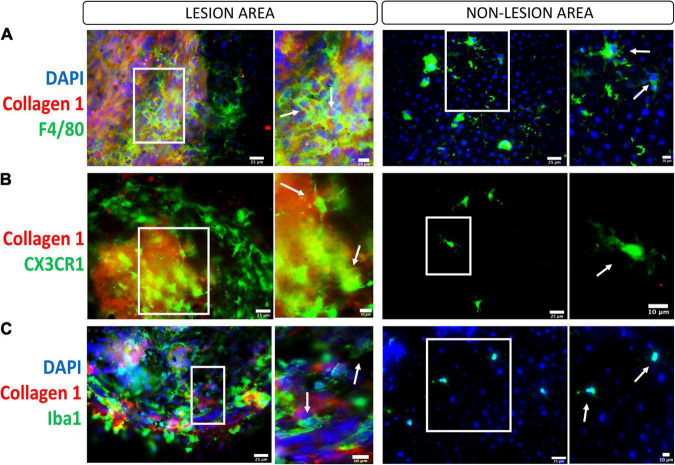
Macrophages/microglia in the subretinal fibrotic lesion. Representative images, and their zoom-in images, of the lesion (left panel) and non-lesion (right panel) area of RPE flat mounts stained for collagen-1, DAPI and F4/80 (**A**, macrophage marker), CX3CR1 (**B**, microglial marker), and Iba1 (**C**, marker for activated microglia and macrophages). Scale bars = 25 μm; Zoom images Scale bars = 10 μm.

Upon the damage of the RPE/Bruch’s membrane complex in laser-induced CNV, microglia and choroidal macrophages are parts of the first wave of infiltrating immune cells (Huang et al., 2013; Liu et al., 2013b). More recently, using single-cell RNA sequencing analysis, Wieghofer et al. (2021) discovered that retinal microglia were the dominant cell subset present in CNV, suggesting an important contribution to CNV progression. However, their role in the development of subretinal fibrosis remains unknown. In the two-stage laser-induced subretinal fibrosis, we detected a large number of F4/80^+^, CX3CR1^+^, IBA-1^+^ cells both inside and around the collagen-1^+^ or fibronectin^+^ fibrotic lesion (Little et al., 2020b; Figure 3). The primary role of infiltrating macrophages is undoubtedly to remove debris and initiate retinal repair, but they can promote subretinal fibrosis during chronic inflammation through multiple mechanisms (Figure 2).

First, macrophages can directly transdifferentiate into myofibroblasts, a process called macrophage-to-myofibroblast transition (MMT). MMT has been shown to contribute to renal fibrosis (Meng et al., 2016). It has been reported that M2, especially CD206^+^ macrophages, rather than M1, undergo the transition (Wang et al., 2017). More recently, a study from our group demonstrated the existence of MMT in subretinal fibrosis (Little et al., 2020b) and further showed that, in addition to TGF-β, the anaphylatoxin C3a could induce MMT (Little et al., 2020a).

Second, macrophages can release pro-angiogenic and pro-fibrotic mediators that can either recruit and activate fibroblasts or induce mesenchymal transition from endothelial or epithelial cells (Wynn and Vannella, 2016; Zhu et al., 2017b). In the case of subretinal fibrosis, infiltrating macrophages may recruit choroidal fibroblasts or circulating fibrocytes to the site of CNV. They may also release pro-fibrotic mediators to induce EMT in RPE cells or endothelial-to-mesenchymal transitions (EndoMT) from choroidal vessels or CNV (Shu et al., 2020; Song et al., 2021).

Third, in response to prolonged tissue damage, macrophages can further promote subretinal inflammation, including the recruitment of other immune cells and complement activation. Macrophages can synthesize various complement components and directly contribute to subretinal complement activation (Luo et al., 2012). Uncontrolled complement activation is believed to drive AMD pathology (Xu and Chen, 2016; Armento et al., 2021). We reported that RPE cells could enhance the expression of complement C3 and complement factor B (CFB) and downregulate complement factor H (CFH) and CD59a expression in macrophages under inflammatory conditions (Luo et al., 2013). Higher plasma level of C3a, C4a, and C5a is related to subretinal fibrosis in nAMD (Lechner et al., 2016), indicative of the involvement of the complement system in subretinal fibrosis.

In humans, there are three functional monocytes subsets (i.e., precursors of macrophages), classical (CD14^+^CD16^–^), non-classical (CD14^–^CD16^+^), and intermediate (CD14^+^CD16^+^) (Wong et al., 2012). We reported that intermediate monocytes in nAMD patients expressed higher levels of HLA-DR (Chen et al., 2016a) and that monocytes from nAMD patients without macular fibrosis, produced higher levels of interleukine-8 (IL-8) and CCL2 (Lechner et al., 2017). The exact subsets of monocytes giving rise to pro-fibrotic macrophages in nAMD remain to be elucidated.

#### Dendritic Cells

Dendritic cells (DCs) are professional antigen-presenting cells involved in tissue homeostasis. Several populations of DCs are present in the eye but the majority of them reside in connective tissues (e.g., cornea, sclera, choroid) with only few in neurons (Forrester et al., 2010). A small number of DCs were reported to be in the peripapillary and peripheral marginal retina in mice (Xu et al., 2007). DC are early responders to retinal injury. Lehmann et al. (2010) reported that CD11c^+^CD11b^+^ DCs responded rapidly to optic nerve injury and light-induced photoreceptor injury. They increased in number and accumulated at the injury site and became MHC-II^+^ (Lehmann et al., 2010), suggesting that DCs are key players in retinal injury and wound healing.

Dendritic cells are known to play a critical role in inflammation-driven fibrosis in multiple organs (Rahman and Aloman, 2013; Bocchino et al., 2021). Ahadome et al. (2016) showed that classical DC contributed to ocular mucosal fibrosis through the retinoic acid pathway in a model of allergic eye disease. Impaired DC maturation can lead to inadequate T-cell response and contribute to organ fibrosis as observed in COVID-19 patients (Borcherding et al., 2021). It has been reported that DCs contribute to scar formation in liver fibrosis and multiple sclerosis directly through secreting metalloproteinase and their inhibitors (Rahman and Aloman, 2013).

**FIGURE 4:**
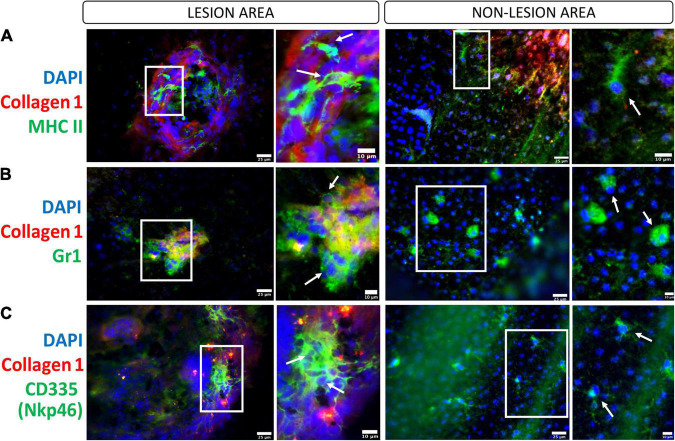
Innate immune cells in the subretinal fibrotic lesion. Representative images, and their zoom in images, of the lesion (left panel) and non-lesion (right panel) areas of RPE flatmounts stained for collagen-1, DAPI and MHCII **(A)**, Gr-1 **(B)**, CD335/Nkp46 **(C)**. Scale bars = 25 μm; Zoom images Scale bars = 10 μm.

The critical role of DC in retinal inflammation has been documented by many studies (Xu et al., 2007; Forrester et al., 2010). In our two-stage laser-induced subretinal fibrosis, a large number of MHC-II^+^ cells were detected inside the lesion (Figure 4A) but their DC identity is unknown. The role of DC in subretinal fibrosis thus remains to be elucidated (Figure 2).

#### Neutrophils

Neutrophils are known to be associated with acute inflammation and are one of the first immune cells recruited to the site of injury or infection. Neutrophil respiratory burst through the NADH oxidase system is essential for efficient pathogen elimination. In addition, neutrophil granules contain various enzymes [e.g., lactoferrin, neutrophil gelatinase-associated lipocalin (lipocalin-2), gelatinase, etc.], which can participate in bacterial killing (Segal, 2005). Although the number of neutrophils declines rapidly after the initial phase of acute inflammation, neutrophil elastase and neutrophil extracellular traps (NETs) are known to critically contribute to inflammation-mediated organ fibrosis (Martínez-Alemán et al., 2017) including lung inflammation and fibrosis in COVID-19 patients (Wang et al., 2020; Figure 2).

The role of neutrophils in inflammation-mediated retinal fibrosis has not been systemically investigated. We and others have shown that retinal inflammation in the CCL2 or CCR2 deficient experimental autoimmune uveitis (EAU) mice is dominated by neutrophils (Sonoda et al., 2011; Chen et al., 2012). Interestingly, inflammation-induced intraretinal fibrovascular membrane is reduced in CCR2 KO (Chen et al., 2012) and CCL2/CX3CR1 double knockout mice (Zhao et al., 2014a). In oxygen-induced retinopathy (OIR), vascular remodeling is associated with neutrophil infiltrating and NETs can remove diseased endothelial cells and remodel unhealthy vessels (Binet et al., 2020). Data from the EAU and OIR studies appear to suggest that neutrophils may promote retinal vascular repair and reduce pathological fibrosis. In nAMD, the circulating level of neutrophils is higher compared to age-matched healthy controls (Niazi et al., 2019). We found that the plasma level of lipocalin-2 is increased in nAMD patients with macular fibrosis (Chen et al., 2020), suggesting a link between dysregulated neutrophil activation and macular fibrosis. In the two-stage laser-induced mouse model of subretinal fibrosis, we detected GR-1^+^ cells around and inside the lesion (Figure 4B). Further studies will be needed to understand the role of neutrophils in inflammation-induced retinal fibrosis.

#### Natural Killer Cells

Natural Killer (NK) cells are cytotoxic lymphocytes critically involved in innate immunity. It has been suggested that NK cells promote angiogenesis and ameliorate fibrosis in the liver and heart (Radaeva et al., 2006). On the other hand, NK cell accumulation could promote chronic kidney inflammation (Turner, 2017) and this has also been seen in severe COVID-19 patients with pulmonary fibrosis (Bi, 2022).

In the eye, infiltration and malfunction of NK cells have been reported in patients with non-infectious uveitis such as Behcet’s disease (Kucuksezer et al., 2015), Vogt-Koyanagi-Harada disease (Levinson et al., 2016), and in viral uveitis (Hamzaoui et al., 1990). CNV is a serious complication of chorioretinitis/posterior uveitis, particularly in the forms affecting the outer retina-RPE-choroid interface (Baxter et al., 2013). The inflammation-induced CNV can become a fibrovascular membrane if remains untreated (Kim et al., 1987; Chen et al., 2012) but the role of NK cells in the development of uveitis-related retinal fibrovascular membrane remains to be investigated. In patients with proliferative DR (Obasanmi et al., 2020) and nAMD (Lechner et al., 2015), the percentage of CD56^+^ NK cells was not altered compared to that in healthy controls. In our two-stage laser-induced subretinal fibrosis, we detected Nkp46^+^ NK cells in the fibrotic lesion site (Figure 4C). A previous study showed that NK cells could produce VEGF when co-cultured with RPE (Hijioka et al., 2008). However, another study showed that human iPS-derived RPE greatly suppressed NK cell activation (Sugita et al., 2018). Further studies are required to understand the role of NK cells in subretinal fibrosis.

#### Mast Cells

Mast cells (MCs) are granulocytes involved, at different levels, in immune responses such as allergy responses, wound healing, angiogenesis, and immune tolerance (Krystel-Whittemore et al., 2016). MCs are particularly abundant within the mucosal and connective tissues of the skin, lungs, guts and are in proximity to small venules and capillaries. Their activation leads to the release of various mediators (e.g., histamine, tryptase, chymase), cytokines and chemokines (Krishnaswamy et al., 2005). MCs have been implicated in the pathogenesis of fibrotic conditions in the liver, kidney, skin, and lung (Overed-Sayer et al., 2014). Mechanistically, MCs can promote inflammation by releasing various vasodilators and proinflammatory mediators, and producing profibrotic factors such as bFGF, PDGF, and TGF-β (Monument et al., 2015). MCs can also activate fibroblasts through cell-to-cell communication *via* gap junction (Yamamoto et al., 2000). MC activation has been observed in COVID-19 patients and is believed to contribute to cytokine storm and related organ damage and fibrosis (Conti et al., 2020).

In the eye, MCs are primarily found in the choroid but absent in the retina (McMenamin, 1997). Elegant studies from Gerard A. Lutty’s group demonstrated that the number and degradation of MC are increased in all forms of AMD including early AMD, GA, and nAMD (Bhutto et al., 2016). They further showed that mast cell-derived tryptase plays a critical role in the development and progression of the GA (McLeod et al., 2017). MC activation and degradation are also reported to contribute to pathological angiogenesis in OIR (Matsuda et al., 2017). In patients with idiopathic epiretinal membrane and idiopathic macular hole, MCs were detected in the *bursa premacularis* (Sato et al., 2019) suggesting that they may be involved in the development of epiretinal membrane. The implication of MCs in subretinal wound healing and fibrosis remains elusive (Figure 2).

#### Eosinophils

Eosinophils are major effectors of the innate immune system and are involved in a range of inflammatory conditions such as hypereosinophilic syndrome or asthma and eosinophilic esophagitis. Activated eosinophils are an important source of pro-fibrotic and proangiogenic factors like TGF-ß, IL-13, CCL-18, FGF-9, VEGF, and VCAM-1 (Wynn, 2008; Aceves, 2014). They are known to play a role in endomyocardial fibrosis (Spry, 1989) and pulmonary fibrosis, including SARS-CoV-2-induced respiratory inflammation and fibrosis (Kim et al., 2021, 19).

In the eye, eosinophils are known to play a role in allergic conjunctivitis (Trocme and Aldave, 1994), and Wegner’s granulomatosis (Trocme, 1991), although little is known about their involvement in ocular fibrosis. Intraocular eosinophils were detected in *Toxocara canis* and *Ascaris suum* infected eyes (Rockey et al., 1979) but not in human CNV (Grossniklaus et al., 2005, 7). Since eosinophils are one of the major sources of pro-fibrotic mediators, further studies will be needed to elucidate their role in retinal fibrosis (Figure 2).

### Adaptive Immunity

#### T Lymphocytes

During inflammation, infiltrating lymphocytes, in particular, T helper cells can influence the healing and scarring process (Zhang and Zhang, 2020). A previous study reported that CD4 T cell depletion decreased wound strength, resilience and toughness; whereas CD8 T cell depletion increased wound strength, resilience and toughness (Davis et al., 2001). The tissue-educated different subsets of T cells can secrete various mediators and growth factors that influence the microenvironment and directly affect the activity of macrophages and myofibroblasts, key cells for wound healing and fibrosis. It is believed that wound healing and fibrosis are orchestrated by Th2 cells, which secrete anti-inflammatory and pro-fibrotic factors such as IL-4, IL-5, and IL-13. These type 2 cytokines induce excessive deposition of proteins crucial for ECM remodeling, including pro-collagens, matrix metalloproteinase, etc. (Kryczka and Boncela, 2015). Th2 cytokines can induce pro-healing M2 macrophage differentiation. The cytokine IL-5 can activate eosinophils to release fibrotic factors IL-13 and TGF-ß (Le Moine et al., 1999). Regulatory T cells can also facilitate wound healing through upregulation of epidermal growth factor receptor expression (Nosbaum et al., 2016). Other T helper cells, including Th22, Th9, Th17, and T regulatory cells are all known to play a role in organ fibrosis (Zhang and Zhang, 2020). For example, CD4 and CD8 T cell accumulation and elevated levels of IL-17 and type 1 cytokines have been observed in severe COVID-19 patients, similar to idiopathic pulmonary fibrosis patients (Wu et al., 2020).

In the chronic phase of EAU, the development of intraretinal fibrovascular membrane is related to higher levels of IL-17 production, CD4 T cell and arginase-1^+^ macrophage accumulation (Chen et al., 2012), suggesting that both Th17 response and M2-type macrophages may play a role (Figure 2).

Previously, we reported that the percentage of CD4, but not CD8 T cells was significantly higher in nAMD patients with macular fibrosis compared to those without macular fibrosis (Lechner et al., 2015). Moreover, the levels of IL-4 were higher in nAMD patients suggesting an activated Th2 response (Yu et al., 2016) although direct evidence supporting the role of Th2 response in macular fibrosis secondary to nAMD is lacking (Figure 2).

#### B Lymphocytes

B cells are mainly involved in humoral immunity by producing antibodies. Compelling evidence suggests that B cells play an important role in inflammation-mediated fibrosis through antibody-independent mechanisms (Shen and Fillatreau, 2015). B cell deficient mice are resistant to silica-induced lung fibrosis (Arras et al., 2006), and carbon tetrachloride-induced liver fibrosis (Thapa et al., 2015). Mechanistically, active B cells can produce cytokines (e.g., TNF-α, IL-9) and chemokines (e.g., CCL7) that shift pro-fibrotic immune response; they can also interact with T cells, macrophages and myofibroblasts promoting fibrosis (Zhu et al., 2017a).

The role of B cells in non-infectious uveitis is well recognized (Smith et al., 2016). B cells have been shown to infiltrate the retina/choroid in choroiditis-related subretinal fibrosis (Kim et al., 1987). B cells were detected in the epiretinal membranes from proliferative DR (Tang et al., 1993). Retinal autoantibodies have been detected in AMD patients (Adamus et al., 2014). In our previous study, we did not observe any significant difference in circulating B cell population between nAMD patients and healthy controls. The number of circulating B cells in nAMD patients with and without macular fibrosis also did not differ (Lechner et al., 2015). The role of B cells in retinal fibrosis remains elusive (Figure 2).

## Conclusion

When the retina or subretinal space suffers from a one-off mild injury, retinal glial cells and the complement system can heal and repair the damage to restore homeostasis. Once the injury is removed, inflammation will resolve, and the damages will be healed by gliosis without significant immune cell infiltration (“cold fibrosis”). However, when subretinal insult persists, circulating immune cells will be summoned leading to chronic inflammation that is executed by active microglia, the complement system, and various infiltrating immune cells (e.g., macrophages, neutrophils, T cells, etc.). The initial phase of immune cell infiltration is dominated by innate immune cells such as neutrophils and monocytes. As the disease progresses to chronic stages, retina-exposed innate immune cells will educate T and B cells in the regional lymph nodes. These educated T and B cells may migrate to the damaged retina and participate in wound healing. Within the retina, they will be further activated by alarmins released from damaged cells with the aim to clean the dead cells, remove debris and promote repair, although their activation will be regulated by remaining neurons and RPE cells. The wound will be filled with myofibroblasts (recruited or transdifferentiated through EMT, EndoMT, and MMT), EMC deposition and infiltrating innate and adaptive immune cells (“hot fibrosis”). Improved knowledge of how the immune cells orchestrate retinal/subretinal wound healing response, in particular, why and how the response is disrupted and/or dysregulated, could lead to the development of new therapeutic strategies to prevent or treat retinal fibrosis.

Future studies should aim to understand the cellular and molecular pathways involved in retinal wound healing, in particular the crosstalk between neurons and the immune system in the healthy and the damaged retina. knowledge of how chronic insult (e.g., oxidative stress in RRD and AMD) breaches the retinal IP, and how it affects the crosstalk between neurons and the immune system will be critical to uncovering molecular pathways underlying dysregulated retinal inflammation during wound healing.

The outstanding questions for developing preventive or therapeutic strategies for subretinal or intraretinal fibrosis include: (1) what are the signals that recruit and retain circulating immune cells in different stages of retinal wound healing? (2) how are the phenotype and function of infiltrating immune cells regulated by the retinal microenvironment at different stages of wound healing? (3) which immune cells are the key drivers of retinal fibrosis and what pro-fibrotic molecules that they produce?

## Ethics Statement

All experiments using mice were performed under the regulation of the United Kingdom Home Office Animals (Scientific Procedures) Act 1986 and the protocols were approved by the Animal Welfare Ethical Review Body of Queen’s University Belfast (PPL2876).

## Author Contributions

MS and HX did the literature search for this review and wrote the manuscript. MS drew Figures 1, 2 with conceptual input from HX. ML-S and MC supervised immunostaining, reviewed, edited, and provided counsel on the manuscript. All authors have read and approved the final version of the manuscript.

## Conflict of Interest

The authors declare that the research was conducted in the absence of any commercial or financial relationships that could be construed as a potential conflict of interest.

## Publisher’s Note

All claims expressed in this article are solely those of the authors and do not necessarily represent those of their affiliated organizations, or those of the publisher, the editors and the reviewers. Any product that may be evaluated in this article, or claim that may be made by its manufacturer, is not guaranteed or endorsed by the publisher.

## Abbreviations

AMD: age-related macular degeneration
BRB: blood retinal barrier
CFB: complement factor B
CFH: complement factor H
CNV: choroidal neovascularization
COVID-19: Coronavirus disease 2019 DCs, dendritic cells
EAU: experimental autoimmune uveitis
ECM: extracellular matrix
EndoMT: endothelial-to-mesenchymal transitions
EMT: epithelial-to-mesenchymal transition
FFG-2: fibroblast growth factor-2
iBRB: inner blood retinal barrier
IL: interleukin
IP: immune privilege
MMT: macrophage-to-myofibroblast transition
nAMD: neovascular AMD
oBRB: outer blood retinal barrier
OIR: oxygen-induced retinopathy
PEDF: pigment epithelium derived factor
PDGF: platelet-derived growth factor
PDR: proliferative diabetic retinopathy
PVR: proliferative vitroretinopathy
RPE: retinal pigment epithelium
RRD: rhematogenous retinal detachment
SARS-CoV-2: severe acute respiratory syndrome coronavirus 2
TGF-β: transforming growth factor-ß
TNF-α: tumor necrosis factor-α
VEGF: vascular endothelial growth factor.
